# Can radiation dose in mammography be further reduced by increasing the image quality?

**DOI:** 10.1186/bcr2973

**Published:** 2011-11-04

**Authors:** D O'Leary, L Rainford

**Affiliations:** 1 University College Dublin, Ireland

## Introduction

Efforts to reduce radiation dose should be *linked to *and *limited by *image quality and the radiation dose should only be lowered to levels *compatible with *image quality for adequate diagnosis; this is optimisation of mammography. Despite this link between image quality and mean glandular dose (MGD), the MGD is proposed only with regard to compression level achieved or population percentile, not image quality required/achieved.

## Methods

A study of symptomatic breast units geographically spread over Ireland collected image quality and radiation dose data. The quantitative and qualitative data were analysed using mathematical modelling and SPSS statistics including ANOVA. High image quality was matched to lowest achievable radiation doses; inadequate images were discarded from the dataset for recommendations of achievable MGD.

## Results

MGDs received by perfect images are significantly lower than the radiation doses received by inadequate images. This is seen in both digital images and film-screen images and for both mammographic projections: digital craniocaudal, *F*(3,978) = 2.841, *P *= 0.37; digital mediolateral oblique, *F*(3,977) = 4.896, *P *= 0.002; analogue craniocaudal, *F*(3,785) = 7.993, *P *< 0.001; analogue mediolateral oblique, *F*(3,783) = 7.961, *P *< 0.001). The mean MGD in mGy required to produce a perfect image in each of the categories: digital craniocaudal, 1.23 mGy; digital mediolateral oblique, 1.28 mGy; analogue craniocaudal, 2.10 mGy; analogue mediolateral oblique, 2.25 mGy.

## Conclusion

The publication of average glandular dose must be linked to image quality achieved and the percentage of inadequate images in the data collection must be explicit. The radiation doses can be lowered further in digital imaging by greater training of radiographers to consistently achieve perfect mammography images.

